# Hematuria as an Early Sign of Multisystem Inflammatory Syndrome in Children: A Case Report of a Boy With Multiple Comorbidities and Review of Literature

**DOI:** 10.3389/fped.2021.760070

**Published:** 2021-10-27

**Authors:** Ana Generalić, Maša Davidović, Ivanka Kos, Kristina Vrljičak, Lovro Lamot

**Affiliations:** ^1^Department of Pediatrics, University Hospital Center Zagreb, Zagreb, Croatia; ^2^General Hospital “Dr. Tomislav Bardek”, Koprivnica, Croatia; ^3^Department of Pediatrics, School of Medicine, University of Zagreb, Zagreb, Croatia

**Keywords:** hematuria, multisystem inflammatory syndrome in children (MIS-C), SARS-CoV-2, kidney, Kawasaki disease

## Abstract

**Introduction:** While the clinical course of SARS-CoV-2 infection seems to be milder or asymptomatic within the pediatric population, growing attention has been laid to the rare complication elicited by virus, multisystem inflammatory syndrome in children temporarily associated with COVID-19 (MIS-C). Published definition and criteria of MIS-C include persistent fever, multisystem involvement, and elevated markers of inflammation, without obvious microbial inflammation or other plausible diagnosis. However, the aim of this case report is to emphasize the diversity of symptoms of MIS-C, beyond the defined criteria.

**Case Presentation:** We present a 10-year-old boy with 8p23.1 microdeletion syndrome and multiple comorbidities who initially came to our attention due to hematuria, persistent fever, rash, and elevated markers of inflammation. Within the next 2 days, his condition worsened despite the broad-spectrum antibiotic therapy. Assuming his past history of SARS-CoV-2 exposure, MIS-C was suspected. A high level of clinical suspicion was further supported by significant clinical features (vomiting, abdominal pain, conjunctivitis, arrhythmia, and mild left ventricular systolic dysfunction with pleural effusion) along with laboratory findings (elevated ESR, CRP, proBNP, D-dimers and fibrinogen, positive IgG SARS-CoV-2 antibodies, and negative microbiological cultures). The patient was given intravenous immunoglobulin (IVIG) and began to show instantaneous clinical and laboratory improvement.

**Conclusion:** Despite numerous reports of MIS-C cases in children, there are still many uncertainties regarding the clinical presentation and laboratory findings, as well as mechanisms beyond this intriguing disorder. In our case, for the first time hematuria is reported as an early symptom of MIS-C. We strongly believe that reporting various manifestations and outcomes in MIS-C patients will lead to improved diagnosis, treatment, and overall understanding of this novel inflammatory condition.

## Introduction

The first experience from the beginning of the COVID-19 pandemic showed that children rarely developed severe or critical illness (1) or die from the infection as compared with adults (2, 3). Nevertheless, since the beginning of the pandemic, multisystem inflammatory syndrome in children (MIS-C), a new phenomenon with temporal association with COVID-19, has become a great concern of parents and pediatricians worldwide (4–6).

The pathogenesis of this syndrome remains largely unknown, but immunological mechanisms and vasculopathy have been implied (7). Literature data are growing on this subject, and although a unique definition and criteria have still not been established, prospective studies and case series have described multiorgan involvement, most commonly including the gastrointestinal, mucocutaneous, cardiac, and respiratory systems (8, 9). Renal involvement is a rather rarely reported manifestation of MIS-C, most commonly presented as acute kidney injury (AKI) in children with a complicated disease course, and seldom as hematuria, proteinuria, and pyuria (Table 1) (10–31). With continued pandemic and increasing awareness of this syndrome among clinicians, it is expected that more cases of MIS-C will be diagnosed and unusual presentations are likely to be seen, while, on the other hand, extensive medical and public attention might result in overdiagnosis of MIS-C, which highlights the need for reporting informative cases (32). Therefore, we describe an unusual case of a boy who initially presented with hematuria, persistent fever, rash, and elevated markers of inflammation, while, within the next 2 days he developed multisystem involvement and met the criteria for MIS-C. Moreover, we performed a literature review of MIS-C patients presenting with renal symptoms. The aim of this case report is to emphasize the diversity of symptoms of MIS-C, beyond the defined criteria.

**Table 1:** Clinical characteristics, treatment modalities, and outcome of MIS-C patients with renal impairment.

**First author (ref. no.)**	**MIS-C Pts**	**Kidney manifestation**	**Treatment**	**Outcome**
Cassim (10)	1	Myoglobinuria, AKI	IVIG, corticosteroids, renal replacement therapy, ICU	Complete recovery
Rodriguez-Smith (11)	19	AKI 6/19	IVIG 17/19, corticosteroids 16/19, anakinra 5/19, antibiotics 14/19, antiviral 1/19, ICU 12/19	NA
Sica (12)	1	AKI	IVIG, corticosteroids	Recovered with new onset hepatic steatosis
Basalely (13)	55	AKI 10/55	Anakinra 49/55, corticosteroid 35/55, IVIG 6/55, remdesivir 1/55, ICU 34/55	AKI resolved in 90%
Duarte-Neto (14)	3	AKI 3/3	IVIG 2/3, corticosteroids 1/3, antibiotics 2/3, antiviral 2/3, ICU 3/3	Death 3/3
Eckard (15)	2	AKI 2/2	remestemcel-L 2/2	Residual hypertension 1/2
Abdel-Haq (16)	33	AKI (common among ICU pts)	IVIG 29/33, infliximab 14/33, corticosteroids 1/33, antibiotics 27/33, remdesivir 2/33, ICU 22/33	Recovered 33/33 (100%), thrombotic complication 1/33 (3%)
Onyeaghala (17)	1	AKI	Corticosteroids, antibiotics, hydroxychloroquine	Complete recovery
Fernandes (18)	69	AKI 17/69	Corticosteroids 32/69, IVIG 41/33, remdesivir 5/69, ICU 44/69	Discharged home 66/69 Death 0/69
Biko (19)	10	Non-obstructing renal calculi 1/10	IVIG 7/10, corticosteroids 6/10, donated plasma antibodies 2/10, ICU 9/10	Discharged home 9/10 Death 0/10
Ozsurekci (20)	7	AKI 7/7	Plasma exchange 7/7, continuous renal replacement therapy 2/7, favipiravir 2/7	Discharged home 6/7 Death 0/7
Diorio (21)	18	AKI 5/18 Proteinuria 12/18	NI	Discharged home 18/18
Nino (22)	1	AKI	Antibiotics, tocilizumab, corticosteroids, ICU	Complete recovery
Greene (23)	1	AKI	Antibiotics, tocilizumab, IVIG, corticosteroids, ICU	Complete recovery
Mahajan (24)	1	Hematuria and pyuria AKI	IVIG, corticosteroids, anakinra, remdesivir, ICU	Discharged with diffuse ectasia in the LAD demonstrated
Lee (25)	1	AKI	IVIG, corticosteroids	Discharged with EFLV shortening (28–32%)
Grewal (26)	28	Variable degrees of hematuria, proteinuria and pyuria, AKI 15/28	ICU 28/28, kidney replacement therapy 8/28	Deaths 0/28
Stefanachi (27)	1	End-stage renal disease	Antibiotics, corticosteroids, hyperimmune plasma, kidney replacement therapy, ICU	Discharged with antihypertensive therapy
Plouffe (28)	1	Transient microhematuria, idiopathic acute renal infarction	Aspirin	Recovered
Garcia-Dominguez (29)	4	Transient AKI 2/4	IVIG 3/4, corticosteroids 3/4, antibiotics 4/4	Recovered 4/4
Blumfield (30)	16	AKI 5/16	Corticosteroids 10/16, IVIG 5/16, anakinra 2/16	Discharged 15/16 Deaths 0/16
Del Greco (31)	4	AKI 4/4	Antibiotics 3/4, IVIG 2/4, enoxaparin 1/4, corticosteroids 4/4	Discharged 4/4

## Methods

A systematic literature search was conducted to identify MIS-C patients with renal involvement. The Scopus and MEDLINE/PubMed databases were searched (from November 1, 2019, to August 30, 2021) by entering the following keywords “MIS-C” and “kidney” according to the published guidance on narrative reviews. The following parameters were noted from the studies including MIS-C patients: renal impairment, treatment, and outcome. Twenty-two articles describing 277 patients with renal manifestation of MIS-C were found during the literature search (Supplementary Figure 1).

## Case Presentation

We present a 10-year-old boy with 8p23.1 microdeletion syndrome who presented to our pediatric emergency department at the end of February 2021, with dry cough and fever lasting for 2 days. Additional medical problems of the patient, associated with microdeletion syndrome, included psychomotor delay, behavioral complications, and complex congenital heart disease that underwent complete surgical correction in infancy. Despite the variety of comorbidities, his underlying medical conditions have been well controlled. His recent medical history included exposure to SARS-CoV-2 infection. The patient's mother, a nurse in a COVID-19 intensive care unit, tested positive for SARS-CoV-2 after onset of symptoms in January 2021. At that time, our patient remained asymptomatic. In the middle of February, he developed dry cough with subfebrile temperature that resolved over several days, but no test for SARS-CoV-2 was performed.

At physical examination, he was febrile and well appearing. He had erythematous maculopapular rash on the right lower leg, while auscultation revealed decreased breathing sound on the right side. Laboratory tests showed neutrophilic leukocytosis, an elevated C-reactive protein (CRP 149.75 mg/l), and hematuria (135 RBC/μl). Initial chest X-ray showed no obvious consolidation or pleural effusion. He was discharged with a diagnosis of acute respiratory infection and recommended to take amoxicillin/clavulanate orally.

The patient returned to our emergency department the following day due to persistent fever, vomiting, prostration, loss of appetite, and abdominal pain, but without significant guarding or peritoneal signs. Due to further elevation of CRP (222.59 mg/l) and progression of hematuria (4,200 RBC/μl) with proteinuria (2+ by dipstick analysis), the patient was admitted to inpatient care. The preadmission screening polymerase chain reaction (PCR) test of SARS-CoV-2 was negative. An abdominal x-ray showed stool burden, while abdominal ultrasound revealed a small amount of free fluid, with no other specific findings. Acute surgical emergency was ruled out by a pediatric surgeon consultant, and constipation was successfully managed with glycerin suppositories and lactulose solution. Further management included intravenous administration of crystalloid solutions and ceftriaxone, but his condition continued to worsen within the next 2 days. He developed bilateral conjunctivitis and irregular heart rhythm. Electrocardiography detected nodal rhythm with ventricular extrasystoles, while echocardiography revealed pleural effusion and mild left ventricular systolic dysfunction (EFLV 46–59%) without coronary artery abnormalities. Moreover, pro-B-type natriuretic peptide (proBNP) was elevated (12,270 ng/l).

**Figure 1:**
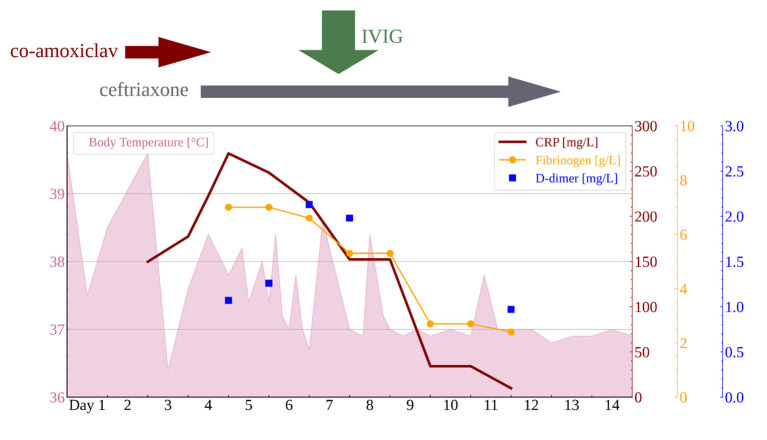
Body temperature and laboratory findings.

The differential diagnosis at this point included sepsis, myocarditis, and Kawasaki disease. Nevertheless, all three were ruled out by negative blood and urine cultures and normal values of troponin I, creatinine kinase, and platelets, while repeated echocardiography showed no increase of coronary arteries. Assuming his past history of SARS-CoV-2 exposure, MIS-C was finally suspected after 5 days of fever. A high level of clinical suspicion was supported by positive SARS-CoV-2 immunoglobulin G (IgG) and rising erythrocyte sedimentation rate (ESR 40 mm/h), CRP (269.5 mg/l), proBNP (12.270 ng/l), D-dimers (2.13 mg/l), fibrinogen (7.0 g/l), and hypoalbuminemia (34.0 g/l), as shown in Tables 2, 3 and Figure 1.

**Table 2:** Clinical, laboratory and imaging findings.

**CLINICAL FINDINGS**	
**Significant clinical findings**	**Other**
**Persistent fever ≥ 48 h**	**Unchanged neurological status**
**Abdominal pain and vomiting**	**No signs of acute abdomen**
**Maculopapular rash and non-exudative conjunctivitis**	**Normal urine output**
**Arrhythmia**	**Normal blood pressure and oxygen saturation**
**COVID-19 exposure prior to the onset of symptoms**	
**IMAGING FINDINGS**	
**Pathological**	**Normal**
**Small amount of free abdominal and perihepatic fluid**	**No signs of an abdominal inflammation**
**New-onset pleural effusion**	**Normal initial chest X ray**
**Left ventricular systolic dysfunction (EFLV 46%–59%)**	**No coronary artery abnormalities**
**Nodal heart rhythm and premature ventricular beats**	
**LABORATORY FINDINGS**	
**Pathological**	**Normal**
**CRP (up to 269.5 mg/l)**	**RBC, Hb, Hct**
**WBC (up to 20.41 × 10^12^/l)**	**Platelets**
**Neutrophilia (up to 84%)**	**PV, APTV**
**ESR (up to 40 mm/s)**	**Procalcitonin**
**Fibrinogen (up to 7.0 g/l)**	**Urea, creatinine**
**D-dimers (up to 2.13 mg/l)**	**C3, C4, CH50**
**proBNP (up to 12,270 ng/l)**	**Ferritin**
**Albumin (up to 34.0 g/l)**	**Troponin I**
**SARS-CoV-2 IgG positive, IgM negative**	**Total protein**
	**IgG, IgA, IgM**
	**AST, ALT, GGT, LDH**
	**SARS-CoV-2 PCR nasal swab negative**
	**Negative microbial samples (urine culture, hemoculture, stool, pharyngeal and nasal swab)**
**URINE EXAMINATION (SPOT)**	
**Dipstick**	**Microscopy**
**Leukocyte esterase up to 2+**	**RBC up to 4,200/mm^3^**
**Protein up to 2+**	**WBC up to 25/mm^3^**
**Ketones up to 1+**	**No urinary casts**
**Urobilinogen up to 1+**	
**Blood up to 3+**	
**Nitrites and glucose negative**	
**URINE EXAMINATION (24 H)**	
**Proteinuria 0.41 g**

**Table 3:** Stepwise pathway to diagnosis.

	**(A)**	**(B)**	**(C)**	**(D)**
	**IDENTIFICATION OF CARDINAL SYMPTOMS AND SIGNS**	**CONSIDERATION OF LIKELY DIAGNOSIS**	**SELECTION OF APPROPRIATE WORK-UP**	**INITIATION OF TREATMENT**
**1**	**Increased fever and inflammatory markers (CRP, ESR, WBC)**	**Bacterial infection**	**Search for source of infection (e.g., urinalysis, chest X-ray, microbiology);** **SARS-CoV-2 PCR**	**Antibiotics**
**NO IMPROVEMENT WITH ANTIBIOTIC TREATMENT**				
**2**	**Persistent fever** **Abdominal pain** **Vomiting** **Rash** **Conjunctivitis** **Pleural effusion** **Arrhythmia** **Hematuria** **Proteinuria (dipstick test)**	**Resistant bacterial infection (e.g., intra-abdominal infection, pyelonephritis)** **Immune-mediated diseases (e.g., sJIA, MAS, Kawasaki disease, MIS-C)** **Glomerulonephritis (primary and secondary)**	**Electrolytes and acid-base status ** **Liver function tests** **Lipid blood test** **Ferritin** **Coagulation** **Troponin I and proBNP** **Urea, creatinine, cystatin C** **24-h urine protein test** **SARS-CoV-2 serology** **ECG** **Echocardiography** **Kidney ultrasound**	**None**
**MIS-C DIAGNOSIS ESTABLISHED**				
**3**	**SARS-CoV-2 IgG**	**Vasculitis**	**Complement**	**IVIG**
	**Elevated d-dimers, fibrinogen and proBNB**	**SLE** **PIGN**		
	**Persistent hematuria**			
	**Persistent proteinuria (24-h urine test)**			
	**Left diastolic dysfunction**			
**COMPLETE RESOLUTION OF SYMPTOMS AND SIGNS**				
**4**	**No relevant symptoms or signs**	**None**	**Follow up urine analysis, CBC, CRP, ESR, urea, and creatinine;**	**None**
			**ANA, ANCA, kidney biopsy (in case of hematuria and/or proteinuria relapse)**	
**NO RELAPS OF SYMPTOMS AND SIGNS**				

During this period, hematuria started to resolve, but low-grade proteinuria persisted (24-h urine protein 0.41 g/dU). Global kidney function tests (urea and creatinine), urine output, liver function test, ferritin levels, complement components (C3, C4), and total complement activity (CH50) were within the normal range during the whole course of the disease. There was no cytopenia or decrease in hematological parameters and no casts in urine sediment. Moreover, there were no episodes of hypotension, a decrease in blood oxygen saturation, and no need for inotrope support (Table 2). Finally, on the morning of the seventh day of illness, IVIG was administered in a single dose of 2 g/kg. Therapeutic effects included prompt downtrend of fever, inflammatory markers, and proteinuria, as shown in Figure 1, along with gradual improvement of left ventricular systolic function (EFLV 65–68%) and complete resolution of pleural effusion within the next few days. After 10 days of ceftriaxone therapy, the patient was discharged from the hospital. Outpatient follow-up after 4 weeks revealed complete normalization of clinical and laboratory findings, with no new symptoms and/or signs after 6 months.

## Discussion

Children with clinical and laboratory findings associated with inflammation have always presented a diagnostic challenge in everyday clinical practice. On top of many established immune-mediated disorders, in the era of the COVID-19 pandemic MIS-C needs to be considered as well, especially in children unresponsive to antimicrobial therapy (Table 3). Nevertheless, despite the growing number of reports in the literature, the full extent of the MIS-C manifestations remains unknown. Conversely, a wide range of reported symptoms and signs makes the diagnostic process of this novel syndrome confusing or delayed. Therefore, early recognition and valid interpretation of characteristic features lead to accurate diagnosis and proper treatment, and eventually to elucidation of the underlying mechanisms.

Although an enhanced understanding of the processes beyond the MIS-C is essential for effective treatment, despite many recent efforts they remain inconclusive. Different pathogenesis traits have been proposed, but the predominant theory is the postinfection antibody-mediated disease (7, 33, 34). However, evidence is mounting that vasculopathy has an important role in MIS-C (33, 34). Evidence supporting this theory incorporates the resemblance of MIS-C and Kawasaki disease, a known vasculitis (35–37). Similar features of these two inflammatory disorders include prolonged fever, increased inflammatory markers, rash, non-exudative conjunctivitis, and mucous involvement (cracked lips, strawberry tongue) (33, 35–37). Despite these resemblances, there are also marked differences in clinical manifestations, such as older age of onset, more frequently observed gastrointestinal involvement, and a more severe disease course in MIS-C, as well as dissimilarities in laboratory findings, such as lymphopenia, thrombocytopenia, and elevated D-dimer levels in MIS-C (33, 35, 37). Comparison of inflammatory cells and 180 plasma proteins in patients with MIS-C and Kawasaki disease revealed that both conditions have elements of hyperinflammation and vasculitis but a different cytokine pattern, suggesting different pathogenesis (33). Other noteworthy characteristics of MIS-C implying vasculopathy is the common presence of coagulation disorders (34, 38, 39). Elevated levels of D-dimer and von Willebrand factor are seen in almost all patients with MIS-C, while fibrinogen levels and prothrombin time are also frequently increased (34, 38, 39). In addition, an intriguing case report has been published, describing intestinal ischemia in a patient with MIS-C presenting with severe abdominal pain and pseudoappendicular syndrome, suggesting intestinal vasculitis (40).

Moreover, it has been suggested that MIS-C could be a severe form of acute COVID-19 infection (7, 34). This theory is supported by the fact that children mostly lack respiratory symptoms and therefore a nasal swab positive for COVID should not be expected. Instead, Rowley et al. proposed PCR analysis on stool samples since children mainly have gastrointestinal symptoms (7). On the other hand, the respiratory mucosal epithelium is most commonly the entry point of virus in adults (41), and therefore, it is fair to mark the lung as a “primary battle zone.” Accordingly, the highest levels of SARS-CoV-2 virus per cell were detected within the respiratory tract. Nevertheless, viral particles were also isolated from many other organs, including the kidney, liver, brain, heart muscle, blood, small intestine, and even sweat glands and skin (41). Many studies reported that SARS-CoV-2 was isolated from the kidney in COVID-19 patients with kidney involvement or coexisting chronic kidney disease (42). Multiorgan tropism and its affinity and affection of the kidney especially indicate that AKI might be a consequence of direct viral toxicity (42). However, it remains unclear if the cell invasion is the sole mechanism responsible for kidney manifestations of COVID-19. Angiotensin-converting enzyme 2 (ACE 2) has been identified as the cell entry receptor for SARS-CoV-2, making the tubular cells that harbor ACE2 especially vulnerable and resulting in tubular damage (43). On the contrary, new cases of collapsing glomerulopathy emerged in patients of African ancestry who are homozygous for *APOL* risk alleles, potentially suggesting other specific molecular mechanisms (44).

The European Renal Association revealed that advanced chronic kidney disease is an independent risk factor for poorer outcome of COVID-19 (45), while *de novo* kidney disease is commonly seen in hospitalized patients with COVID-19 as well, especially in those critically ill (46). The available literature most commonly describes renal involvement in children with MIS-C as acute renal failure or AKI (4, 39, 47–50). The prevalence of renal involvement varies greatly depending on the studied population (4, 39, 47–49). Two French studies, in which complicated MIS-C cases needing intensive care were enrolled, described a prevalence of 59 and 70%, respectively (4, 48), while a large US cohort of 570 MIS-C patients reported AKI in 18% of patients (42, 47); an Iranian study reported renal failure in 29% of patients (49). In most studies, the course of renal failure was not elaborated in detail (38, 47, 49). In the US study, kidney injury was sought a complication of severe MIS-C (47); in the Iranian study a consequence of high levels of ACE2 in the kidney (49). Since renal failure is present more frequently in COVID and MIS-C patients needing intensive care, circulatory shock may be an important contributor to renal failure development (4, 48, 51).

Additionally, among 277 MIS-C patients with renal involvement identified during our literature search, the most commonly reported manifestation was AKI (Table 1). Compared to patients without AKI, it was more frequent among patients who had cardiac dysfunction, required inotropic support, and ICU admission (26). Accordingly, pathogenesis of AKI appears to be predominantly pre-renal (26). On the other hand, severity of kidney dysfunction in patients with AKI did not correlate with degree of cardiac dysfunction (26). Beside AKI, other renal manifestations or urinalysis results in MIS-C patients were described only in few articles, reporting variable degrees of hematuria, proteinuria, and pyuria (21, 24, 28). Interestingly, abnormal urinalysis as indicator of renal parenchymal injury was present in a significant proportion of AKI patients (21, 24). Furthermore, proteinuria was a predominant symptom in MIS-C patients who also met the clinical criteria for thrombotic microangiopathy (TMA) (21). TMA is a clinical syndrome defined by the presence of hemolytic anemia, thrombocytopenia, and organ dysfunction due to endothelial cell damage and formation of microscopic blood clots in capillaries and small arteries. Soluble C5b9 (Sc5b9), a biomarker of complement activation and TMA, was elevated in patients with SARS-CoV-2 disease, as well as in MIS-C patients (21). Consequently, increased plasma levels of the terminal complement complex (sC5b9) suggest that complement activation and thrombotic microangiopathy are prevalent in COVID-19 and MIS-C patients (21). Although many of the described patients beside IVIG required an additional treatment with glucocorticoids, antibiotics, biological agents, and even plasmapheresis and renal replacement therapy, in many complete recovery was reported (10, 17, 22, 23, 28, 29).

It remains unknown whether hematuria and proteinuria were really absent in the published cohorts or they were underreported. Although hematuria and proteinuria might have been neglected in MIS-C patients, they are rather common manifestations of adult COVID-19 infection (51–53), ranging from 7 to 63% of patients for proteinuria (51) and 26.7% (52) and 40.9% (53) for hematuria. Histopathological analysis of renal tissue of COVID patients revealed viral fragments in the cytoplasm of proximal tubules as well as podocytes, which could potentially explain proteinuria (51). Another common finding in adults is obstruction of glomerular lumen and peritubular capillaries by erythrocyte aggregates (51). In children, hematuria and SARS-CoV-2 infection were described in a 9-year-old girl who presented with increased temperature, cough, and gross hematuria, which resolved spontaneously (54). SARS-CoV-2 was isolated not only from her nasal swab but also from her urine sample (54). Another case describes a 13-year-old boy who presented with purpuric rashes, mild hematuria, elevation of serum IgA, and biopsy-confirmed leukocytoclastic vasculitis secondary to asymptomatic SARS-CoV-2 infection (55). This is in line with the notion that hematuria is most commonly associated with vasculitis syndromes such as immunoglobulin A (IgA) nephropathy or anti-neutrophil cytoplasmic autoantibody (ANCA)-associated vasculitis (56). Furthermore, recent findings suggest that gross hematuria can damage the glomeruli and lead to AKI (56).

In our patient, there were no signs of kidney function deterioration, hypotension, or need for inotropic support, although a moderate cardiac dysfunction was noted. Moreover, he had no signs of TMA or complement dysfunction. Besides, he had a complete and persistent resolution of all symptoms and signs following only IVIG therapy, without further need for glucocorticoid treatment. Therefore, no additional laboratory tests or invasive procedures were performed. Consequently, limitations of this case presentation are lack of ANA and ANCA screening and lack of kidney biopsy results, which restricts the conclusion about the possible underlying mechanisms in our patient. Nevertheless, of note is that ANCA-associated vasculitis after COVID-19 was described in a few patients presenting with hematuria, proteinuria, and AKI (57, 58). Hence, SARS-CoV-2 infection is suspected to be the trigger of subsequent development of immune-mediated disorders, which prompts a long follow-up in susceptible patients.

In summary, the pathogenesis of renal involvement in COVID-19 is probably multifactorial, and proposed mechanisms include direct infection of renal parenchyma *via* ACE2 receptors which are highly present in kidney tissue (43, 52, 59), microvascular injury caused by cytokine storm and/or hypercoagulability (25, 43, 51, 59), and circulatory shock (4, 48, 49).

To the best of our knowledge, this is the first detailed case of hematuria and concomitant proteinuria in an MIS-C patient. Since additional investigations and follow-up revealed no other pathological finding, we are confident that hematuria in the presented patient was a part of MIS-C. This is further supported by the fact that our patient had no sign of hypovolemia, which could have potentially caused kidney damage, and prompt response to IVIG therapy. Since proteinuria and hematuria have resolved, we did not proceed with further investigations.

We report this unusual MIS-C case because the COVID-19 pandemic is still ongoing, and the number of MIS-C cases will probably continue to grow. Moreover, it is not false to predict that other possible pandemics in the future might also cause similar symptoms. We strongly believe that unusual and yet undescribed clinical presentations are useful for the practicing clinicians, especially with diseases like MIS-C, where early recognition is essential for treatment and good outcomes. Moreover, detailed clinical reports can also inform further research on pathogenesis and possible treatment options. Finally, we consider our case to be another piece of evidence tipping the scale toward the underlying process in MIS-C.

## Data Availability Statement

The original contributions presented in the study are included in the article/Supplementary Material, further inquiries can be directed to the corresponding author.

## Ethics Statement

Written informed consent was obtained from the individuals for the publication of any potentially identifiable images or data included in this article.

## Author Contributions

LL conceptualized the work. AG and MD drafted this manuscript. IK, KV, and LL revised this manuscript. All authors were involved in the clinical management of the patient and read and approved the manuscript for submission.

## Conflict of Interest

The authors declare that the research was conducted in the absence of any commercial or financial relationships that could be construed as a potential conflict of interest.

## Publisher's Note

All claims expressed in this article are solely those of the authors and do not necessarily represent those of their affiliated organizations, or those of the publisher, the editors and the reviewers. Any product that may be evaluated in this article, or claim that may be made by its manufacturer, is not guaranteed or endorsed by the publisher.

## Supplementary Material

Supplementary Figure 1 The schematic overview of the articles on MIS-C patients with renal impairment included in the literature research.

## Abbreviations

ACE2: angiotensin-converting enzyme 2
AKI: acute kidney injury
ANCA: anti-neutrophil cytoplasmic autoantibody
COVID-19: coronavirus disease-2019
CRP: C-reactive protein
EFLV: left ventricular ejection fraction
ESC: erythrocyte sedimentation rate
ICU: intensive care unit
IVIG: intravenous immune globulin
IgA: immunoglobulin A
IgG: immunoglobulin G
MIS-C: multisystem inflammatory syndrome in children
proBNP: pro-B-type natriuretic peptide
PCR: polymerase chain reaction
RBC: red blood cell
SARS-CoV-2: severe acute respiratory syndrome coronavirus 2
TMA: thrombotic microangiopathy.
