# Supplementary figures and images for: Hematuria as an Early Sign of Multisystem Inflammatory Syndrome in Children: A Case Report of a Boy With Multiple Comorbidities and Review of Literature
Source: Front Pediatr. 2021 Oct 27;9:760070. doi: 10.3389/fped.2021.760070 (PMC8579050; doi:10.3389/fped.2021.760070)

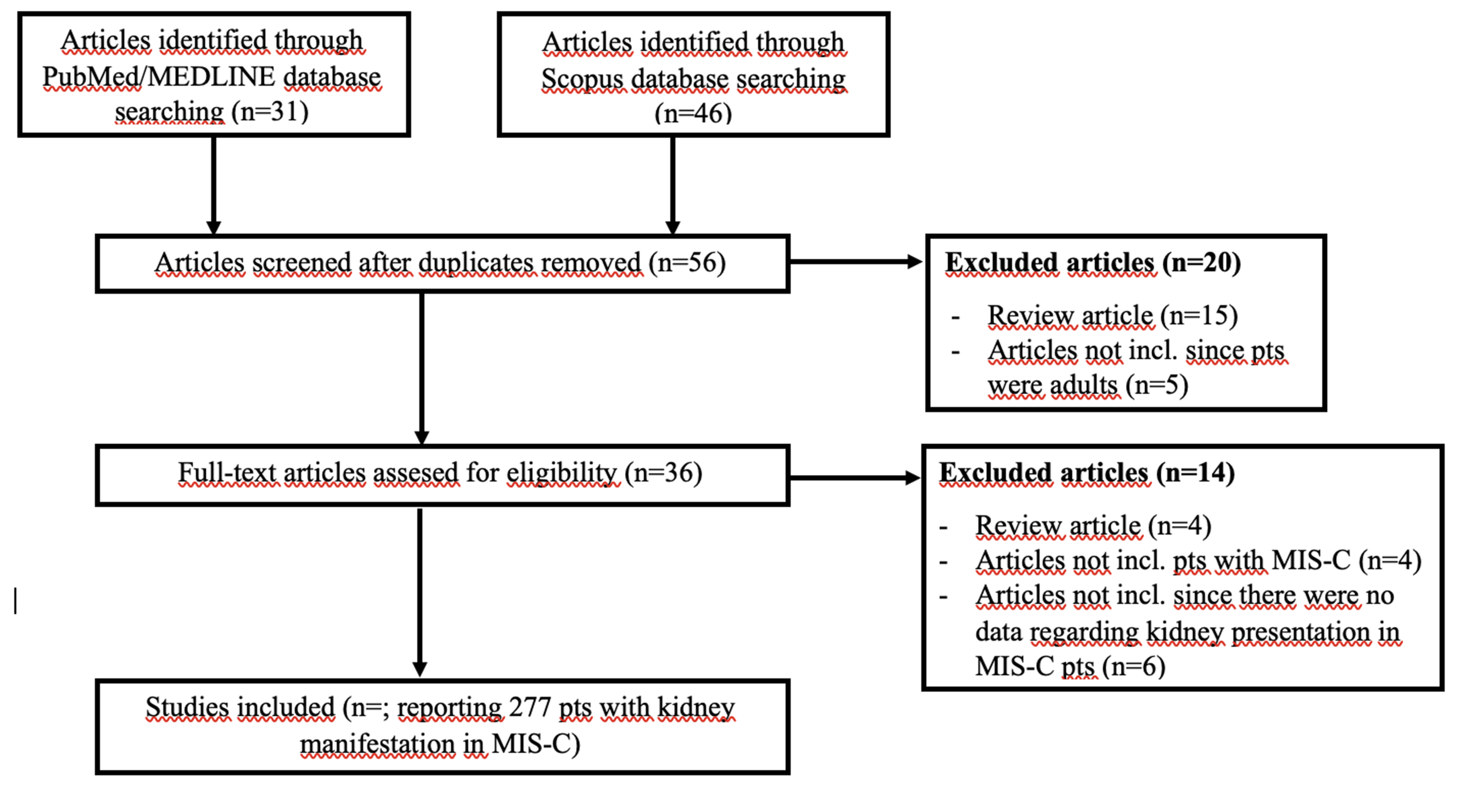

Supplement: Supplementary Figure 1 — The schematic overview of the articles on MIS-C patients with renal impairment included in the literature research. [file Image_1.PNG]
